# Evidence for optimal semantic search throughout adulthood

**DOI:** 10.1038/s41598-023-49858-9

**Published:** 2023-12-18

**Authors:** Jeffrey C. Zemla, Diane C. Gooding, Joseph L. Austerweil

**Affiliations:** 1 Department of Psychology, Syracuse University, Syracuse, NY USA; 2 Department of Psychology, College of Letters and Science, University of Wisconsin-Madison, Madison, WI USA; 3 Department of Psychiatry, SMPH, University of Wisconsin-Madison, Madison, WI USA; 4 Department of Medicine, Division of Gerontology and Geriatrics, SMPH, University of Wisconsin-Madison, Madison, WI USA

**Keywords:** Human behaviour, Cognitive ageing

## Abstract

As people age, they learn and store new knowledge in their semantic memory. Despite learning a tremendous amount of information, people can still recall information relevant to the current situation with ease. To accomplish this, the mind must efficiently organize and search a vast store of information. It also must continue to retrieve information effectively despite changes in cognitive mechanisms due to healthy aging, including a general slowing in information processing and a decline in executive functioning. How effectively does the mind of an individual adjust its search to account for changes due to aging? We tested 746 people ages 25 through 69 on a semantic fluency task (free listing animals) and found that, on average, retrieval follows an optimal path through semantic memory. Participants tended to list a sequence of semantically related animals (e.g., lion, tiger, puma) before switching to a semantically unrelated animal (e.g., whale). We found that the timing of these transitions to semantically unrelated animals was remarkably consistent with an optimal strategy for maximizing the overall rate of retrieval (i.e., the number of animals listed per unit time). Age did not affect an individual’s deviation from the optimal strategy given their general performance, suggesting that people adapt and continue to search memory optimally throughout their lives. We argue that this result is more likely due to compensating for a general slowing than a decline in executive functioning.

## Introduction

Most organisms engage in costly search for food, nutrients, and other resources necessary to live. Organisms that search their environmental niche in an efficient manner have a competitive advantage over their peers in survival and reproduction. As a result of natural selection, many organisms exhibit behavior that is consistent with the goal of optimizing search: maximizing the rate at which resources are encountered^1–3^.

To search efficiently, organisms store knowledge of their environment and retrieve it to guide their search^4^. The human mind is faced with an analogous search problem: it stores a vast amount of semantic knowledge, and people must search through that knowledge efficiently to find contextually relevant information. Individuals who search their semantic memory efficiently are at an advantage compared to those who do not. While most people no longer need to forage for food in the wild, semantic search is still ubiquitous in everyday life. Across physical, visual, and mental search, researchers have found that the mind searches for information in a way that is close to optimally adapted to the environment^5–10^. For example, empirical learning and forgetting curves in memory^11^ reflect the actual probabilities that we encounter information in the real world^12^. One limitation of these studies is that they focus on younger adults, and do not address how performance changes with age. Stored knowledge and retrieval mechanisms change across the lifespan^13^, but to date little research has explored whether the mind compensates for these changes to preserve optimal semantic search throughout adulthood.

In this paper we test whether semantic search is optimal (relative to an individual) in the semantic fluency task (listing as many animals as possible in a fixed time limit), and whether search remains optimal or becomes suboptimal with age. Prior research has found that in aggregate, people search memory optimally in this task, listing animals in a sequence that maximizes the total number of animals retrieved in the time period^7^. We expand on this by using a different analytic technique to obtain a participant-level measure of optimality, and assess whether adherence to optimal search changes across the lifespan. The experiment focuses on adults who are young or middle-aged (approximately 25-50 years old), with fewer participants above the age of 60. The results have implications for how executive functioning and processing speed declines that commonly accompany aging^14,15^ may affect search performance.

### Optimal search and the semantic fluency task

The computational problem of how to efficiently retrieve semantic information can be likened to foraging in a physical environment^16^. In physical environments resources are often distributed in clumpy patches rather than uniformly. Within a patch, a forager faces diminishing returns: the rate of retrieving resources declines over time until the patch has been exhausted. For example, berries are distributed on berry bushes (patches). The more berries one has found on a bush, the longer it will take to find the next berry on that same bush. As a result, it is not efficient to exhaust the resources within a patch entirely. Rather, it is better to leave the patch at some point in search of a new patch that is more plentiful. This also has a cost: searching for a new patch takes time and effort, and no resources are retrieved until a new patch is found. The structure of the environment invokes a classic exploration-exploitation dilemma: How long should a forager exploit the current patch, and when should they search for a new patch? Understanding the cognitive mechanisms that guide explore-exploit decisions has proven crucial to explaining behavior across a variety of psychological domains^17,18^.

**Figure 1:**
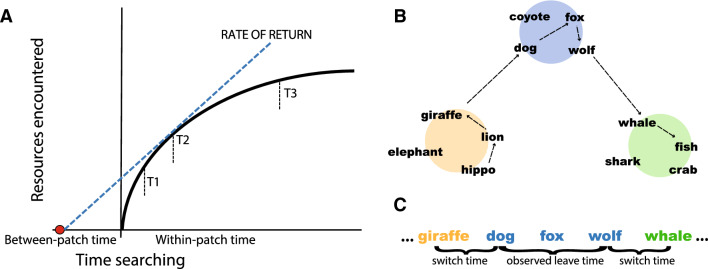
(**A**) Resources are encountered with diminishing returns within a patch. A forager who leaves the patch at T2 (optimal leave time) maximizes the average rate of return, whereas a forager who leaves earlier (T1) or later (T3) does not. The optimal solution is to leave a patch when the local rate of return drops below the average rate of return across all patches, i.e., when the rate of return line lies tangent to the curve. (**B**) Mental search through semantic memory resembles a forager searching over semantic patches. (**C**) Performance in the semantic fluency data can be measured by the response times that span semantic patches (switch times) and the time spent within patches (observed leave times).

In the case of physical search, the solution to this problem is to switch between exploring and exploiting resources in a way that maximizes the rate of encountering resources. If the rate of encountering resources within a patch and the time cost of exploring for a new patch are known, the optimal behavior is to leave a patch when the local rate of return within a patch drops below the average rate of return across all patches. This optimality result is called the marginal value theorem^1^ and can be used to assess whether search is optimal. See Fig. 1A.

Analogously, semantic search resembles foraging over semantically related patches of information. In the semantic fluency task, participants list items from a semantic category (typically *animals* or *foods*). A robust finding is that people will list items in semantically related clusters (i.e., patches;^19^). For example, one might list *dog, fox, wolf* in sequence (all canines) before switching to a different semantic cluster like *whale, fish* (all aquatic animals). See Fig. 1B. In spatial foraging, search time increases with the physical distance that is traversed. In semantic search, inter-item response times are a useful proxy for semantic distance, as the time needed to mentally move from one concept to another is proportional to their semantic similarity^20,21^.

Previous work has found that response times in the semantic fluency task are qualitatively consistent with optimal search of semantic memory. Inter-item response times within a semantic cluster tend to be smaller than the mean response time, while response times that span cluster boundaries tend to be larger than the mean response time^6,7,22^. This pattern suggests that, at minimum, people do not search within a semantic patch for longer than is optimal. In the current study, we quantify adherence to optimal search using the marginal value theorem both at the group level and at the individual level to measure the degree to which people are optimal. Throughout the paper, we use the term “optimal” to refer to whether the timing of cluster switches adheres to the marginal value theorem, i.e., whether a participant switches clusters in a manner that maximizes the number of animals listed given the rate of retrieval within a semantic cluster and the average time needed to switch clusters. While there are other ways that optimality can be construed, our use is consistent with optimal foraging both in the ecology literature and in the semantic retrieval literature^1,3,6,7^.

A different approach to investigating mental search is to explore the many different cognitive strategies that can be used to retrieve concepts from semantic memory^13,23,24^. For example, one might visualize walking through a zoo, or list animals alphabetically. While this work is informative, it is orthogonal to whether people switch clusters optimally in the semantic fluency task. This is because the marginal value theorem is agnostic to the explicit cognitive strategy or retrieval algorithm: It provides a computational-level analysis of behavior, rather than an algorithmic one^25^.

### Age-related decline and semantic retrieval

Many aspects of semantic memory and retrieval change with age. Adults continue to acquire new semantic information with age, evidenced by their larger vocabularies^26,27^, and the mental organization of that knowledge changes throughout life^28–31^. Changes in the quantity or structure of stored semantic knowledge may contribute to retrieval difficulties in older adults^32^. For example, older adults experience more retrieval failures than younger adults when asked to recall a word given its definition, and are slower to name the word on successful attempts^33^. Older adults also experience more tip-of-the-tongue states when recalling low-frequency words^34^, and list fewer exemplars from a semantic category under time constraints^19^. These findings show that aging is associated with declines in the ability to explicitly retrieve semantic information.

Why is semantic retrieval impaired with age? One proposal is that it is due to impaired executive functioning^15,35,36^. In the semantic fluency task, clustering and switching may reflect two distinct components of search^7,19^. Clustering is largely driven by automatic, associative processes, in which new responses are generated based on their semantic similarity to the previous response. Switching is associated with executive functioning processes, and allows individuals to guide search towards concepts that are semantically unrelated to the previous response. In support of this account, neuropsychological studies have found that individuals with frontal lobe lesions (associated with executive functioning deficits) switched clusters less often^37^. Additionally, the number of responses generated is correlated with measures of executive functioning like operation span^38^.

We discuss the *executive functioning hypothesis* in which declines in executive functioning selectively affect the timing of cluster switches, but do not impact the timing of within-cluster search^19^. Under this hypothesis, an individual might take longer to switch clusters but experience no change in the rate of listing items within a cluster. This can result in sub-optimal search when evaluated by the marginal value theorem, because a failure to disengage from a cluster results in spending too much time within a cluster. It is worth noting that some work has cast doubt on the selectivity of executive functioning in cluster switching^39–43^. For example, Mayr and Kliegl^42^ compare performance on a standard fluency task to an alternating fluency task (listing items from two categories in an alternating fashion). An age by condition interaction (larger switch costs for older adults in the alternating condition) is expected if executive functioning is selective to switching. The authors find no evidence of this and conclude their results are “not consistent with the idea of a general, age-related switching deficit.” Our definition of the executive functioning hypothesis differs from this alternative theory in that our hypothesis assumes executive functioning demands are larger for switches than non-switches.

A second proposal is that semantic retrieval is impaired due to a general slowing of cognitive processing that accompanies age^14,44^, which is also supported by prior research. The number of responses generated in semantic fluency is correlated with processing speed (measured using a shared variance component derived from multiple tasks, including the digit symbol substitution task and trail making task)^45,46^. We explore the *processing speed hypothesis* that semantic retrieval slows by a fixed factor, but does not selectively affect within-cluster search or cluster switches. Under this hypothesis, declines in processing speed lead to a proportional increase in response times for all responses generated, regardless of whether they span a cluster boundary or not. This hypothesis predicts that adherence to optimal search should not vary with age, because the predictions of the marginal value theorem are scale invariant.

In this study, we test whether adherence to optimal semantic search in the fluency task varies with age using the marginal value theorem. If processing speed is the primary determinant of age-related semantic deficits, then the optimality of semantic search should not vary with age. However if executive functioning plays a special role in cluster switching, then people may become less optimal as they age.

## Methods

### Participants

We recruited 746 participants for our study using Amazon’s Mechanical Turk and e-mail lists distributed through community organizations. Prior to recruitment and data collection, this study was approved by the University of Wisconsin-Madison Institutional Review Board (protocol 2018-1223). All methods were performed in accordance with the relevant guidelines and regulations. Informed consent was obtained from all subjects.

Data from 527 participants were analyzed (see exclusion criteria below). These participants had a mean age of 38.1 years old (range 25–69, sd 9.5). Participants were not binned into age groups. We use the terms “younger” and “older” participants to refer to participant ages in this range relative to each other (i.e., a 50 year-old is an “older” participant in our sample despite not being “old” in the colloquial sense). 256 participants (48.6%) were female, 270 (51.2%) were male, and 1 participant was transgender. Most participants identified as either white (64%) and/or Black/African American (16%). 515 participants (97.7%) reported that English was their native language (monolingual or multilingual). See Table 1 for additional demographic information.

**Table 1:** Demographics of the participants after exclusionary criteria were applied.

Demographic	N (%)	Demographic	N (%)
Age		Income	
25–29	91 (17.3%)	Under $10,000	19 (3.6%)
30–34	137 (26.0%)	$10,000–$19,999	51 (9.7%)
35–39	112 (21.3%)	$20,000–$29,999	76 (14.4%)
40–44	66 (12.5%)	$30,000–$39,999	79 (15.0%)
45–49	52 (9.9%)	$40,000–$49,999	52 (9.9%)
50–54	33 (6.3%)	$50,000–$74,999	120 (22.8%)
55–59	18 (3.4%)	$75,000–$99,999	52 (9.9%)
60–64	11 (2.1%)	$100,000–$150,000	50 (9.5%)
65–69	7 (1.3%)	Over $150,000	14 (2.7%)
Gender		Undisclosed	14 (2.7%)
Female	256 (48.6%)	Education	
Male	270 (51.2%)	Grammar school	1 (0.2%)
Transgender	1 (0.2%)	High school diploma	
Race/ethnicity		or equivalent	62 (11.8%)
American Indian		Some college	133 (25.2%)
or Alaskan Native	6 (1.1%)	Associate’s degree	41 (7.8%)
Asian$$^{*}$$	70 (13.3%)	Bachelor’s degree	219 (41.6%)
Black, African, Caribbean,		Master’s degree	58 (11.0%)
or African-American	87 (16.5%)	Doctorate degree	1 (0.2%)
Latino	52 (9.9%)	Professional degree	11 (2.1%)
Middle Eastern	4 (0.8%)	Undisclosed	1 (0.2%)
White	339 (64.3%)		
Prefer not to answer	3 (0.3%)		
Multi-ethnic	42 (8.0%)		

*Denotes all Asian individuals including Indian or Pakistani.

### Procedure

The participants took part in a large personality survey of attitudes and experiences, however this survey is not part of the present investigation and is not discussed further. Interspersed within this group of surveys were three trials of the semantic fluency task^47^. In each trial, participants were asked to “List as many animals as you can think of” in three minutes. Participants typed each response into a text field and pressed Enter after each response. After pressing Enter, the response faded from the screen (fading animation lasted 100ms). This procedure was designed to minimize the possibility of previously generated responses cueing subsequent responses. Participants were instructed to not list any animal more than once within a list but could repeat the same animal across lists. The median time to complete the entire study (including unrelated surveys) was 35.4 minutes. Mechanical Turk participants were compensated with a cash reward, while community participants received a Starbucks gift card of equal value.

A time limit of three minutes per fluency trial was used in order to ensure sufficient data was collected from each participant to estimate their retrieval curve. While one minute is perhaps the most common, there is no standard time limit for the task. Other research has employed time limits that vary from thirty seconds^48^ to five minutes^24^. However, increasing the time limit provides diminishing returns (fewer animals listed per unit time), which makes collecting a large quantity of data per participant difficult. To compensate for this, we used three trials of the fluency task per participant^49,50^ which allows us to collect more data than would be possible from a single, nine minute trial.

### Data analysis

The data were pre-processed to ensure participants followed instructions. Fluency lists were removed from the dataset if: the participant did not hit Enter after each response (resulting in the absence of response times); the participant listed fewer than five animals (an arbitrarily low threshold to make sure participants attempted the task in earnest); or the participant listed three or more intrusions (non-animals) across all three lists. Participants who did not complete three valid lists (for the reasons above, or due to attrition) were removed from the dataset. After pre-processing, the dataset contained 1581 lists from 527 participants.

Each response in the dataset was assigned to one or more semantic categories using SNAFU, a software tool for automatically coding fluency data^51^. For example, the response *dog* is a member of both the Pets category and the Canine category. SNAFU assigns category labels using a pre-existing dictionary of animals and their categories. The dictionary we used is an amalgam of several animal taxonomies previously used in the animal fluency literature^7,19,51^ and contains 30 categories of animals. While it is not an exhaustive taxonomy, Zemla et al.^51^ found that it reliably agrees with fluency data that is manually coded for clusters. The dictionary we use is available in the Supplementary Materials. Each animal was assigned to at least one animal category. If a response did not share any category with the previous response, it was marked as a semantic cluster switch. Otherwise, the transition was marked as a continuation of the previous semantic cluster.

Optimal search was quantified using the marginal value theorem^1^, which stipulates that optimal search is achieved by leaving a cluster when the rate of return within a cluster drops below the average rate of return across all clusters. In the context of the semantic fluency task, an individual can maximize their rate of listing animals if they stop listing animals from a given cluster when the rate of listing animals in that cluster drops below the average rate of listing animals (across all clusters). Three key pieces of information are needed for this calculation: (1) the average between-cluster switch time, (2) the average empirical (observed) cluster leave time (i.e., the average time spent in a cluster), and (3) a retrieval curve, indicating the average number of animals listed at any point in time within a cluster.

The between-cluster switch time was measured as the inter-item response time between the last item of one cluster (e.g., *giraffe*) and the first item of a new cluster (e.g., *dog*). The empirical cluster leave time was measured as the time between the first item of a cluster (e.g., *dog*) and the last item of that same cluster (e.g., *wolf*). See Fig. 1C. The optimal cluster leave time was found by calculating the rate of return at any time in a cluster (i.e., the average number of animals listed at time *t* since entering a cluster, divided by the sum of the switch time and *t*), and choosing the time *t* that maximizes this rate of return.

**Figure 2:**
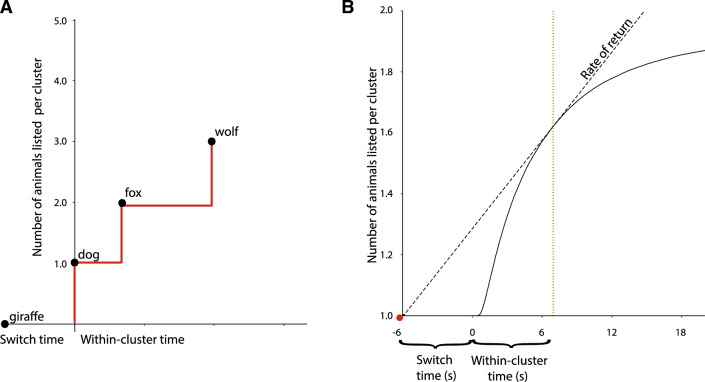
(**A**) A retrieval curve for a single cluster of one participant, starting from the last item of the previous cluster (giraffe) and continuing through the last item of the current cluster (wolf). (**B**) A retrieval curve constructed from averaging all 27,736 clusters in the data. After generating the first animal in a new cluster (at 0s), the rate at which participants list new animals starts to decline (solid black curve). The average rate of listing animals, from the last animal in the previous cluster (red dot) to the last animal of the current cluster (dotted vertical red line), is shown by the dashed diagonal line. This rate is maximized if participants leave the current cluster at 6892ms (dotted vertical green line), whereas participants leave a cluster on average at 6994ms (dotted vertical red line). Given the resolution of this figure, the optimal leave time and empirical leave time virtually overlap (absolute deviance = 102 ms).

In total, 27,736 clusters were identified in the dataset. The retrieval curve for a single cluster in the data can be depicted as a step function; an example is shown in Fig. 2A. By averaging over the step functions of all clusters in the dataset, we can depict the average retrieval curve over the entire dataset (Fig. 2B). Following the convention of Stephens and Krebs^3^, we plot the average switch time (5926ms) to the left of the graph origin and within-cluster time to the right of the graph origin.

The data were analyzed in multiple ways. First, the behavior of the sample was assessed to see whether people search optimally in aggregate. This was followed by an individual-level analysis that allows us to test whether search behavior changes with age. Finally, the group-level analysis was repeated for each fluency trial to examine whether adherence to optimal search changes with practice. 

## Results

Participants listed a mean of 33.73 animals per list (range 5–90, sd 12.25). The mean number of animals generated per participant was not correlated with age ($R = -.01, p =.77$). Additionally, age was not correlated with average cluster size (number of animals in a cluster), average number of cluster switches, cluster switch rate (number of switches per item), or mean response time (all $|R| < .04$, all $p > .46$). Here and in subsequent analyses, response times were not log-transformed. Statistical significance did not change when they were log-transformed, except where noted.

Participants spent an average of 6994ms listing items in a cluster before leaving. Given the average retrieval curve and average switch time, the rate of return is maximized with a cluster leave time of 6892ms (absolute deviance = 102ms). This result suggests that in the aggregate, people are remarkably close to optimal in their cluster switching behavior: When listing animals, people switch between clusters in such a way that maximizes the total number of animals generated.

We further assessed whether this result could have been due to chance alone by resampling the data to simulate new fluency datasets. To construct each simulated dataset, we permuted the response times in the dataset. This method of simulating datasets preserves the pattern of cluster switches across the data (i.e., the sequence of animals and cluster switches remained the same), but results in a new empirical and optimal cluster leave time for each simulated dataset. We repeated this procedure 1,000 times and measured the absolute deviance from optimal search for each simulated dataset. We calculated the distribution of deviances from optimal search across simulated datasets (mean absolute deviance = 1980ms, minimum absolute deviance = 829ms). This does not overlap with the empirical deviation from optimal search (102ms). As such, our result is highly unlikely to be observed by chance alone.

### Individual behavior

**Figure 3:**
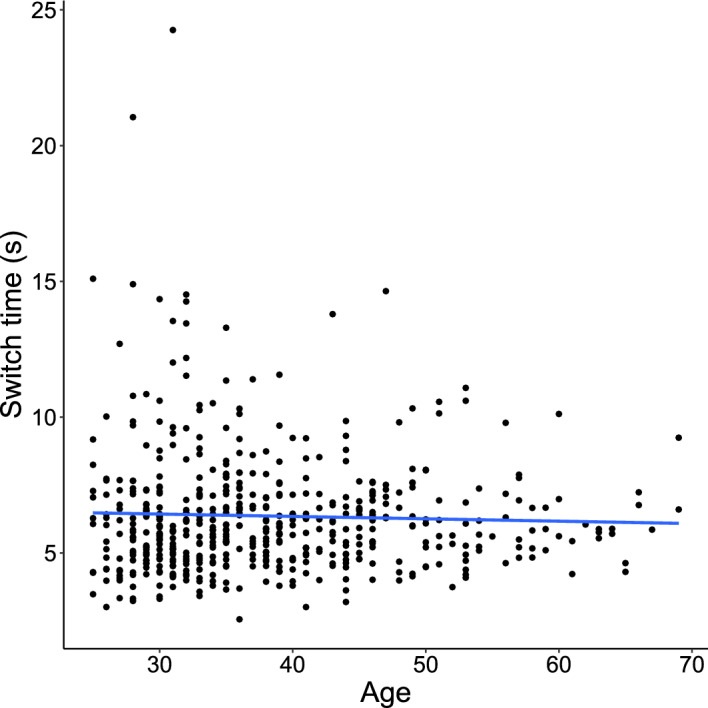
We found no difference in the between-cluster switch times with age. Each point denotes the average between-cluster switch time for a single participant.

**Figure 4:**
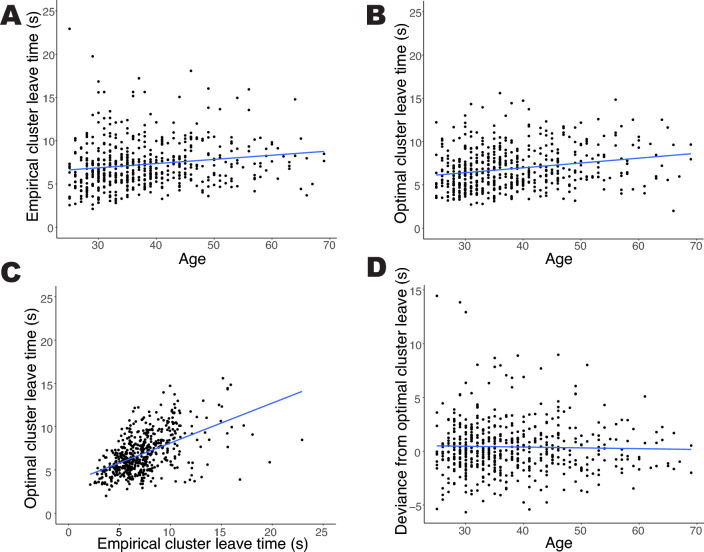
As people age, they do (**A**) and should (**B**) search within a cluster for longer. Optimal cluster leave times correlated significantly with empirical cluster leave times (**C**). We found no difference with age in exhibiting optimal search (**D**).

Between-cluster switch times did not vary by age, $p =.4$ (Fig. 3). However, the amount of time spent in a cluster before leaving increased with age, $r(525) =.17$, $p = .0001$ (Fig. 4A). Optimal cluster leave times increased with age as well, $r(525) =.22$, $p <.0001$ (Fig. 4B), and optimal leave times and empirical leave times were strongly correlated, $r(525) =.53$, $p <.0001$ (Fig. 4C). As a result of increases in both optimal and empirical leave times, adherence to optimal policy did not vary with age ($p =.5$; Fig. 4D). A corresponding Bayes Factor of 0.128 indicates substantial evidence that age does not affect adherence to optimal policy. We use a Bayesian statistic here because one a priori hypothesis predicts that age has no effect on optimality. This finding is consistent with declines in processing speed, which predicts that adherence to optimality does not vary with age. It is more difficult to explain with the executive functioning hypothesis, which predicts that only switch times would vary with age and subsequently optimality would vary with age. This supports processing speed as the primary determinant of age-related changes in semantic fluency.

**Figure 5:**
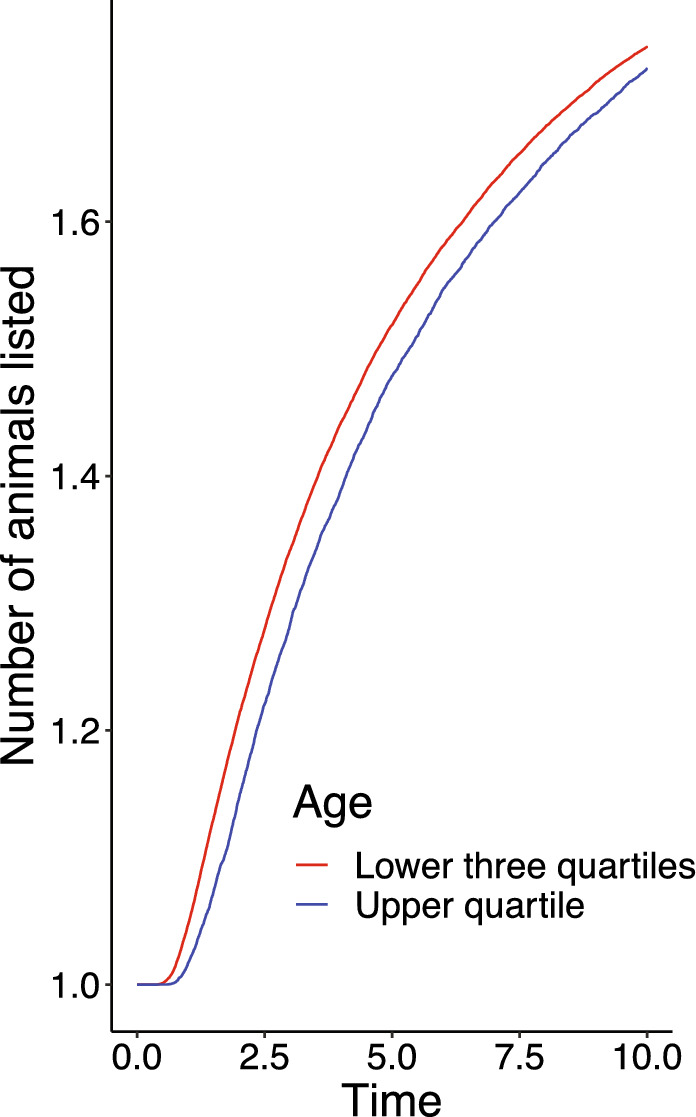
The rate of listing animals within a cluster over time was steeper for younger adults (red line) compared to older adults (blue line). For visualization purposes, we operationalized older adults as the upper quartile of participant ages (44–69) relative to younger participants in the lower three quartile (25–43).

Older adults retrieved animals from memory at a slower rate, with a lower average rate of listing animals within clusters (i.e., a shallower slope of line tangent to the individual’s within-cluster retrieval curve) , $r(525) = -.09$, $p =.035$, reflecting, a shallower retrieval curve for older adults (Fig. 5). It may seem surprising that the rate of within-cluster search varies with age, while the total number of responses does not. However this demonstrates that the rate of within-cluster search is not the sole determinant of performance on the task. In addition, one’s ability to switch clusters at the optimal time *given* this change in retrieval rate is critical to performance. The change in the within-cluster retrieval rate with age is precisely why the marginal value theorem suggests that older adults should spend more time in a cluster (Fig. 4B).

Each individual had a relatively small number of clusters in their data (mean = 52.6, range = 16–123 across all three lists). Deviance from optimal leave times were expected to be larger on average for individuals compared to the group-level analysis, due to the law of large numbers. Deviance from optimal leave time was negatively correlated with the number of clusters in an individual’s data. Additional resampling simulations revealed that deviance from optimal leave time approached zero as the number of clusters increased, but (as expected) not when the response times were permuted. As a result, examining individual behavior without correcting for the number of clusters in each participant’s data (such as Fig. 4) can be misleading. We performed additional simulations to correct for this.

To examine whether participants were more optimal than would be predicted by chance, we conducted a permutation analysis. Mirroring the group analysis, we constructed simulated fluency datasets for each participant by permuting the response times of that participant (1000 times for each participant). On average, participants were 1777ms from optimal, compared to 3209ms in the simulated data. 80% of participants had a deviance from optimal that was lower than the mean of their simulated dataset. This result is not expected by chance alone, $p <.001$ by binomial test.

We also tested whether adherence to optimal search improved performance in the fluency task. For each participant, we calculated the total number of animals listed across the three trials of the task. Performance should improve with both a higher rate of listing animals within a cluster, and a lower deviance from optimal cluster leave times. Using multiple regression we found that both within-cluster rate of return, $\beta = 926300$, $t(522) = 20.43$, $p <.001$, and deviance from optimal cluster leave times, $\beta = -.003$, $t(522) = -4.24$, $p <.001$, were significant independent predictors of the total number of animals listed by each participant (multiple $R^2 =.49$). We also tested a model that included an interaction term (within-cluster rate of return times deviance from optimal cluster leave time). While the interaction term was not significant ($p =.14$), we still observed a main effect for both factors (both $p <.001$). When response times were log-transformed, we observed a significant interaction but not a main effect.

### Trial-level analysis of behavior

One limitation of the above analysis is that the data are collapsed across all three semantic fluency trials. The repeated nature of the task leaves open the possibility that mental search operates differently across the three trials. On the second and third fluency trials, short-term episodic memory of animals retrieved in previous trials may affect how responses are generated, rather than being reflective solely of semantic retrieval. Here, we explore this possibility by analyzing the data from each of the three fluency trials separately.

**Figure 6:**
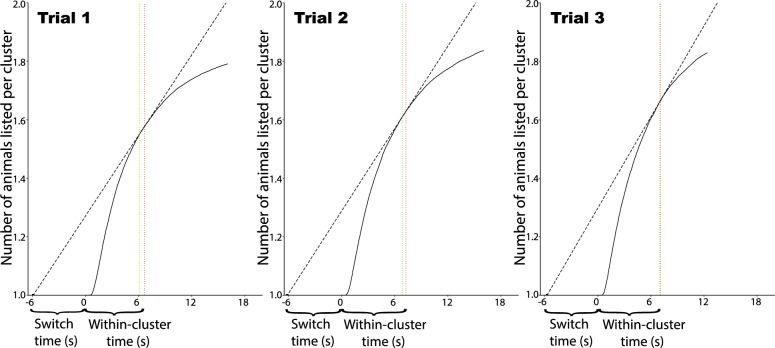
Optimal leave times (green dotted lines) are shown relative to empirical leave times (red dotted lines) for each of the three fluency trials. Leave times are close to optimal for each trial, though the deviance from optimal is largest on the first trial and smallest on the last trial.

In the first trial, where performance is driven solely by semantic retrieval, participants searched each cluster an average of 6663ms, whereas the rate of return is optimized switching at 6073ms. While search is still close to optimal (absolute deviance = 590ms), the deviance is not as small as when aggregating over all three trials. On the second trial, behavior was closer to optimal (empirical leave time = 7267ms; optimal leave time = 6892ms; absolute deviance = 375ms), and even more so on the third trial (empirical leave time = 7073ms; optimal leave time = 7008ms; absolute deviance = 65ms). See Fig. 6.

There are several possible explanations for this. One possibility is that short-term memory for animals generated on previous trials facilitate retrieval at an optimal rate. However, another interpretation is that participants become more familiar with the task with practice and adopt better meta-cognitive strategies for when to switch clusters. The data were also analyzed separately for each trial per participant. Many (but not all) of the results mirror those reported in the Individual Behavior section above, and additional information regarding these analyses can be found in the Supplementary Material.

## Discussion

The marginal value theorem^1^ was originally proposed to evaluate whether animals search for food and other resources optimally, given the patchy structure of their environment. Here, we used the same theorem to evaluate whether people are optimal in semantic search. Using data from a semantic fluency task, we find strong evidence that semantic retrieval adheres to the marginal value theorem both in aggregate and at an individual level. Our work builds on previous research that has also found evidence of optimal foraging in the semantic fluency task^6,7^. It differs in that we employ a new methodological approach to analyzing fluency data that allows for an individual-level measure of optimality and, using this measure, we demonstrate that adherence to optimality does not vary with age. These results add to the growing body of literature that emphasizes the importance of applying computational tools from optimal foraging to improve our understanding how the mind works^17,52^.

Throughout adulthood, people acquire new knowledge and the structure of our semantic knowledge changes. Despite this, we find no evidence that people search less optimally as they age. Although the amount of time spent searching within a cluster increased with age, this is exactly what the marginal value theorem indicates people should do if the rate of retrieval within a cluster decreases. Accordingly, prolonged search within a cluster may represent a compensatory mechanism of the aging mind, as opposed to an age-related impairment in the ability to switch clusters.

We evaluated our results in the context of two theories of age related decline: the executive functioning hypothesis and the processing speed hypothesis. Our results are more consistent with the processing speed theory, though neither captures the behavior perfectly. Under this theory, semantic retrieval slows by a fixed factor which should not impact adherence to the marginal value theorem.

Though optimal search was preserved with age, we also found response time differences for only some components of the task, but not all: average response times and cluster switch times did not change with age, while within-cluster times slowed. This selectivity is inconsistent with the processing speed hypothesis, but not in a way that is compatible with the executive functioning theory, which predicts a selective deficit in switching (i.e., increased switch times) and a change in adherence to optimal search with age. Our results did not support either of these predictions, so we interpret our results as being less compatible with the executive functioning hypothesis. Other factors that change with age, such as memory search demands^32^ likely play a role as well.

### Limitations and future directions

Our work has several limitations. Foremost, we treat the between-cluster switch time and within-cluster rate of retrieval as fixed and well characterized by their averages. In reality, these quantities may change throughout the task: with each encountered cluster, there are fewer animal clusters known to the participant. This may cause between-cluster switch times to increase and within-cluster retrieval rates to decrease. Treating these quantities as dynamic may lead to a better characterization of retrieval behavior, but is more computationally complex. Still, our work provides a benchmark for evaluating more complex models of search, and provides insight into how the mind solves the explore-exploit dilemma central to mental search^17^ under a narrower set of circumstances.

We discuss our results in the context of two hypotheses central to the aging literature, the executive functioning and processing speed hypotheses. However, we did not independently measure these constructs using secondary tasks, nor did we collect measures of vocabulary size that may influence retrieval during the semantic fluency task^38^. Related work has benefited from controlling for lexical or cognitive abilities^53^.

We have shown that people search optimally throughout most of adulthood, but our participant sample does not equally represent all age groups. Our sample skews younger and contains few participants over the age of sixty. Previous research has found that while there are changes in performance on the semantic fluency task throughout adulthood, the sharpest declines occur at later ages (e.g., 70+ years)^54,55^. This may help to explain why some of our results differ from the prior literature. For example, we do not find commonly reported age-related differences in the number of responses generated or average cluster size. However, declines in the 40-60 age range (which is more representative of the older adults in our sample) are less pronounced or sometimes not observed^53,56,57^. The demographic differences in samples have implications for theory as well. For example, Hills et al.^53^ found that semantic retrieval is affected more by changes to executive functioning than processing speed, in contrast to our findings, but their sample had a median age of 68 and a maximum of 99 years old. We caution against generalizing our findings to others who are not representative of our sample demographics (e.g., those 70+ years old). Future work could fill this gap by assessing optimal foraging patterns in a wider age range and in a longitudinal rather than cross-sectional study.

In our data, we denote a cluster switch as any two adjacent responses that do not share a taxonomic category, also known as an associative cluster switch^58^ or fluid cluster switch^51^. However, several methods for demarcating clusters have been used in the literature, including human annotation^59^, word-embedding approaches^7,60,61^, and a single-category cluster approach^19^. We have not verified whether our results hold using alternative methods.

It is important to note that we do not make claims about how people choose to search on an explicit or conscious level. For example, people may choose to list animals alphabetically, or by visualizing themselves walking around a zoo^24^, but our analyses are theoretically neutral regarding this. Rather, our use of the term “optimal” refers to the fact that the timing of cluster switches maximizes the total number of animal listed given the within-cluster retrieval rate, consistent with previous work in the optimal foraging literature, both in ecology and in semantic retrieval^1,3,6,7^. Future work could explore whether instructing participants to engage in a specific cognitive retrieval strategy^13^ affects whether search is optimal.

Finally, we provide evidence for optimal retrieval only in animal naming. Animal naming is the most commonly used category for the semantic fluency task, and is used in both research and clinical settings^62^. However, many semantic categories have been explored in the literature (e.g.^48^) and it is possible that our results do not extend to all semantic categories. Future work should explore whether these results extend to retrieval in other semantic categories (e.g., tools) and to other variants of the fluency task such as phonemic fluency (e.g., listing words that begin with a particular letter).

### Conclusions

We provide an analogy between animal foraging and mental search, and borrow a mathematical framework from the former to evaluate the latter. In the animal foraging literature, the foraging environment is directly observable. In contrast, the semantic representation that we use for mental search is not observable. Though techniques have been developed to estimate semantic representations^49,63^, they still rely on making inferences from behavioral data. As a result, it is difficult to differentiate between competing accounts of how optimal search is implemented in the mind.

We provided a normative analysis of semantic retrieval at the level of an individual and demonstrated that the mind is nearly optimal given the goal of maximizing retrieval rate. How might the mind accomplish this? Broadly, there are two mechanisms that could produce this behavioral pattern: we call them the *efficient retrieval hypothesis* and the *efficient representation hypothesis*.

Under the efficient retrieval hypothesis, people engage in strategic search over a clustered semantic representation^7,61,64^. The decision to switch clusters is guided by a metacognitive process in which people keep track of how long it takes to mentally switch between semantic clusters and the rate of return within the current cluster. With this information, the mind could monitor the rate of retrieval within a cluster and “decide” to switch clusters when the local rate of return drops below the average rate of return across all clusters.

Alternatively, the efficient representation hypothesis suggests that the mind has developed an efficient way of organizing semantic knowledge. Under this hypothesis, an individual’s semantic representation adapts to exhibit optimal search behavior with a simple retrieval process, such as a random walk or Levy flight^6,65,66^, even though the same process does not necessarily produce optimal search behavior under other representations. Previous evidence has suggested that we may use preferential attachment to integrate newly learned concepts within our existing semantic knowledge^67^, which can result in highly efficient small-world-like semantic representations^68^. Under this account, people do not need to possess any metacognitive awareness of clusters or switching. Rather, these constructs may be epiphenomenal—observable in the data, but not resulting from any real psychological mechanism associated with “switching” as previously proposed^19^.

Prior work has found evidence that is consistent with each of these hypotheses. In support of the efficient retrieval hypothesis, there is ample evidence that executive functioning plays a role in semantic fluency and this is commonly (though not always) attributed to strategic search^19^. Recent work has found a unique neural signature associated with switching but not clustering^61^ as well as ramping activity during within-cluster search that could encode rate of retrieval necessary to guide switching behavior. Computational models of the fluency task that assume a special role for cluster switching also provide a good fit to behavioral data^7^.

In support of the efficient representation hypothesis, there is growing evidence that one’s semantic representation changes with age^28–30^. For example, clusters within a semantic representation become more sparse^31^, which may explain why within-cluster search slowed with age for participants in the experiment. Other work has shown that semantic representations may become more modular with age^69,70^. If executive functioning plays a selective role in cluster switching^19,61^, one might expect age-related declines in executive functioning to disrupt efficient retrieval. The persistence of optimal foraging across the lifespan may be taken as evidence that representational changes counteract a decline in executive functioning. In other words, perhaps optimal foraging is preserved with age because of these structural changes, rather than in spite of them. Computational models of the semantic fluency task have also been proposed that do not assume a special role for cluster switching and predict optimal foraging behavior (^6^; though see^71^).

Still, the two hypotheses are not mutually exclusive, as semantic representation and retrieval processes can interact in complex ways^72,73^. Isolating the contributions of representation and process in semantic search has proved difficult^74,75^, making it hard to provide strong support in favor of the efficient retrieval or efficient representation hypotheses. Future empirical contributions combined with advancements in cognitive modeling and neuroimaging may lead to predictions that disambiguate these theories.

## Data Availability

Supplementary material including all data and code is available at https://osf.io/yc625/.
