# Author Correction: Evidence for optimal semantic search throughout adulthood

**DOI:** 10.1038/s41598-025-93617-x

**Published:** 2025-03-26

**Authors:** Jeffrey C. Zemla, Diane C. Gooding, Joseph L. Austerweil

**Affiliations:** 1 Department of Psychology, Syracuse University, Syracuse, NY USA; 2 Department of Psychology, College of Letters and Science, University of Wisconsin-Madison, Madison, WI USA; 3 Department of Psychiatry, SMPH, University of Wisconsin-Madison, Madison, WI USA; 4 Department of Medicine, Division of Gerontology and Geriatrics, SMPH, University of Wisconsin-Madison, Madison, WI USA

Correction to: *Scientific Reports* 10.1038/s41598-023-49858-9, published online 18 December 2023

The original version of this Article contained an error in Figure 4, panel C, where the y-axis label “Optimal cluster leave time (s)" was incorrectly given as “Empirical cluster leave time (s)”. The original Figure 4 and accompanying legend appear below.

**Fig. 4:**
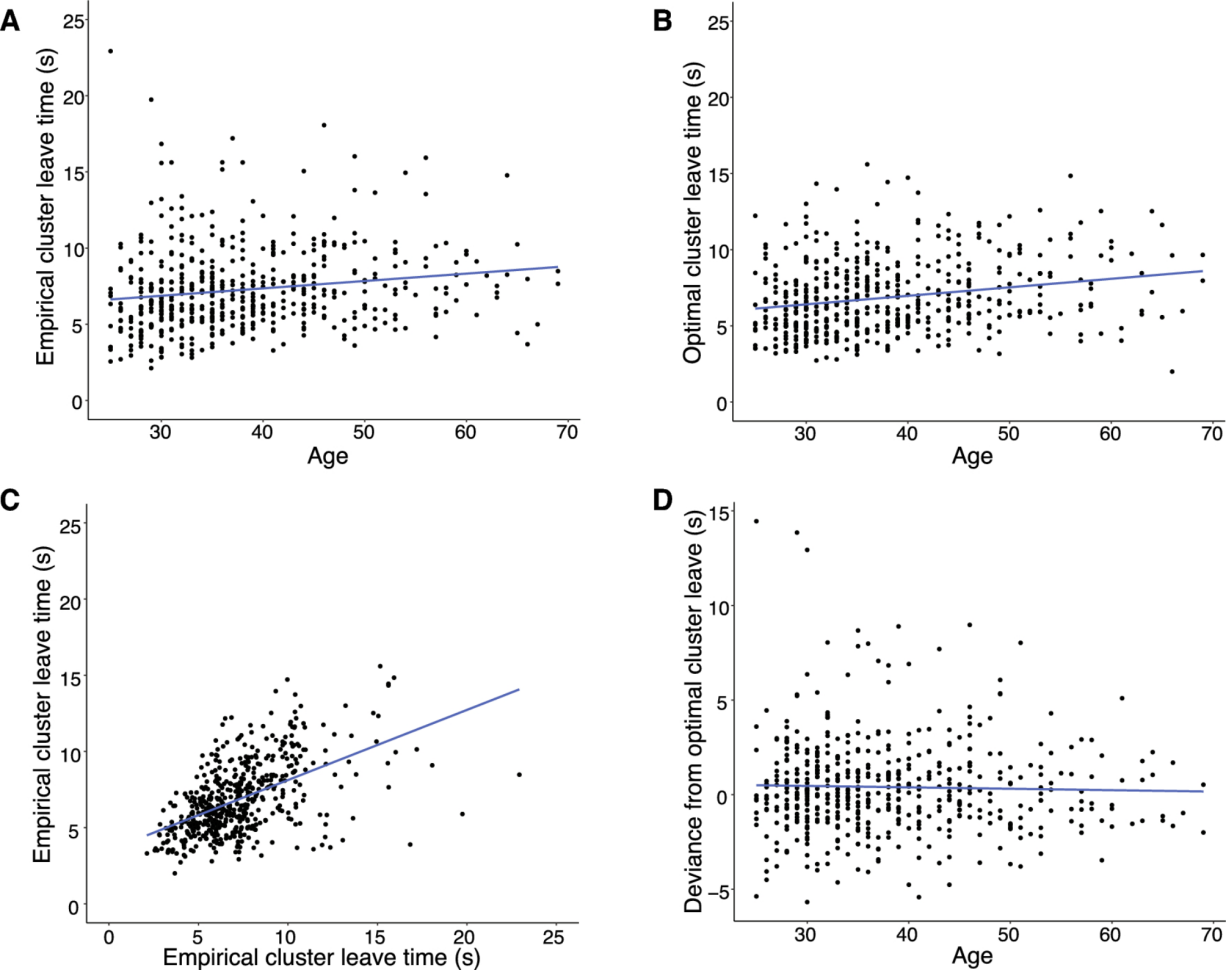
As people age, they do (**A**) and should (**B**) search within a cluster for longer. Optimal cluster leave times correlated significantly with empirical cluster leave times (**C**). We found no difference with age in exhibiting optimal search (**D**).

The original Article has been corrected.

